# The mirage of domestic violence during COVID-19 pandemic in Nepal

**DOI:** 10.7189/jogh.12.03049

**Published:** 2022-07-16

**Authors:** Dilip Bhandari, Prajwal Neupane, Masaharu Tsubokura, Tianchen Zhao, Sampada Gaire, Masazumi Fujii

**Affiliations:** 1 Department of Neurosurgery, Fukushima Medical University School of Medicine, Fukushima City, Japan; 2 Department of Gastrointestinal Tract Surgery, Fukushima Medical University School of Medicine, Fukushima City, Japan; 3 Department of Radiation Health Management, Fukushima Medical University School of Medicine, Fukushima City, Japan; 4 Lumbini Medical College, Palpa, Nepal

One of the social impacts of COVID-19 is domestic violence in developing as well as developed countries. Domestic violence (DV) and homicide has dramatically increased in developing countries during country-wide lockdowns [1]. Countries with well-developed helpline services have received a significant number of reports during lockdowns [2,3]. Das & Das [4] argue that domestic violence throughout the pandemic increased due to stay-home policies, which were a measure for the prevention of COVID-19. DV and COVID-19 were thus viewed as “twin public health emergencies” [4], as lockdowns resulted in victims being forced into an environment with abusers.

Nepal, despite having response policies to prevent DV against females through different national (Maiti Nepal, WOREC, among others.) and international (UNESCO, UNFPA, UN Women, and so on.) organizations, has reported an increment in domestic violence in recent years. 1355 cases of DV were reported from July 16, 2010 to July 15, 2011(1 year), increased to 14 232 reported cases in 10 years (July 16, 2020 – July 15, 2021) [5,6]. According to the Nepal police, 18 879 criminal cases that targeted women, children, and senior citizens were reported in Nepalese fiscal year 077/078 (July16, 2020 – July 15, 2021). The crime against women, children and senior citizens were categorized as rape, attempt to rape, polygamy, child marriage, witchcraft, abortion, untouchability, unnatural intercourse, child sexual abuse, and domestic violence. The highest number 14 232 cases of domestic violence have been reported on Nepalese fiscal year 077/078 with 2532 rape cases being the second on the list and 27 abortion cases being the last. This shows that on average, more than 75% of crime against women, children and senior citizens are of DV [7]. According to the UN special rapporteur in 2018, there were 446 total cases of murder in 2017, wherein 149 females were killed due to gender-based violence. More than half of these were cases of females being murdered by an intimate partner as a form of DV, making it the pronounced cause of violent death in Nepal in 2017 [8]. According to the non-governmental organization Women’s Rehabilitation Center (WOREC) of Nepal, in 2018, domestic violence was the highest reported category of crime against women at 65% among all reported cases. According to earlier reports, 76% of the violence suffered by women was attributed to husbands, while 24% was attributed to family members. Women suffer verbal, physical, and mental abuse and suffer mistreatment and unfair treatment related to their dowry, their inability to give birth to male child or being infertile after years of marriage. In addition, women are denied basic needs and are thrown out of their homes [9].

The total number of reported domestic violence cases during the one-month period before the pandemic lockdown in Nepal in 2020 was 1094, with Province 2 having the highest number of cases, at 313. Province 2 had a higher number of domestic violence cases against women related to dowry and giving birth to a female child than the other seven provinces [8]. After the first month, from the time the lockdown started, the number of cases decreased to 104, which was a 90.49% decline. The cases reported from Province 2 dropped to 15 (95.2% decline). Interestingly, this contrasts with the reported cases of suicide during this time, with a 6% increase in suicide among women (DV is considered as a major risk factor for suicide [10]. The decrease in the number of reported DV cases would have been caused by the lockdown itself; they would have been unable to report due to being trapped at home with abusive family members or partners. A report from IresearchNet’s section on criminal justice shows that only one third of DV cases are reported. Reported cases are, thus, considered just the tip of the iceberg when discussing DV [11], making an abundance of unreported cases extremely likely. This can be inferred from the data because, while the number of reported DV cases during the first month of the lockdown was low, the number of suicide cases by women was 648, with the most number of cases being recorded in Province 2, at 121 [12].

Sujita Thakur, a 26-year-old woman married for 10 years and a resident of Lahan district of Province 2 in Nepal, went missing on June 3, 2021, which was during the second wave of the COVID-19 lockdowns. Her husband and his family accused her saying that she eloped with another man. However, Sujita’s father was not convinced by the eloping story and her leaving a 5-year-old son. He wanted a thorough investigation and went to report the case, but he was rejected by the police, who believed Sujita’s husband. Their neighbours and other locals alleged that her husband’s family had tried to kill Sujita 2 years ago, but this was not reported anywhere. They strongly suspected foul play when they noticed an area with traces of long hair in the Thakur family’s rice field. Believing that this might be where Sujita’s body was buried, they demanded that the police dig the area. The officers found some parts of her body on June 27, 2021. After an investigation was launched following this evidence, her husband, father-in-law, and brother-in-law confessed that they killed her because her father could not give dowry as per their demand. The remaining body parts were found inside the newly constructed toilet tank near her house.

**Figure:**
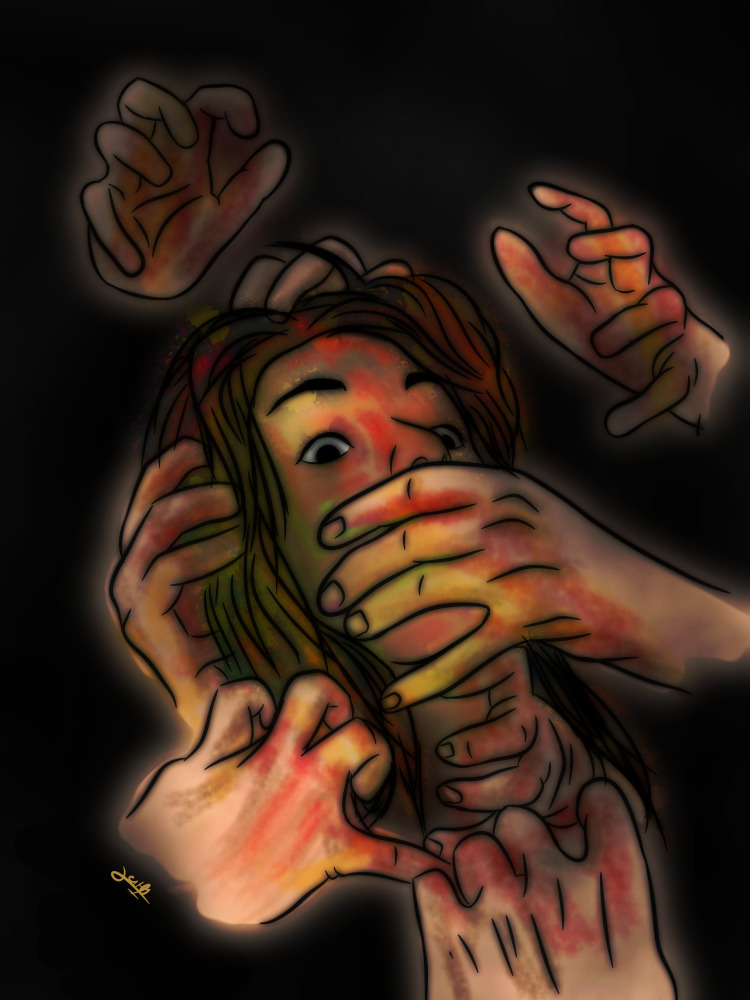
Photo: The image is from Dr Simran Kc and Ritesh Poudel (Hree design and art, used with permission; https://www.instagram.com/p/CM8vJUHBNoJ/?igshid=YmMyMTA2M2Y=).

According to women rights activists, many women do not trust the justice system because their cases were overseen casually, which was the case with Sujita. Women would rather file complaints to non-governmental organizations, resulting in a significant difference between official data and anecdotal evidence [13]. A project from Saferworld funded by USAID –“Community Initiatives for Common Understanding” conducted research on mechanism of justice in Nepal on August 2015 points out rude attitude of police, persistence political interferences in judiciary procedures, less participation of women in political and socio-economical sectors due to strong patriarchal norms as some major factors for distrust in the governmental organizations in Nepal [14].

In several countries, counselling helplines through telephone or texting services are provided by many organizations. A few helplines have been set up in Nepal where women and children can access the centres (**Table 1**). However, most of the women cannot reach safe shelters due to lack of awareness, social stigma, fear of abusers, and so on. Public education, awareness, and programs targeting for building individual commitment to fight against issues of domestic violence is in immediate need. A person from the local community in each municipality can be appointed to relay information to helpline centres, this will also help to reduce the problem with language barrier. Alternatively, helpline centres should involve locals so that victims feel comfortable enough to share their experiences. In Sujita’s case, before her death in 2021, if the neighbours who had witnessed her being abused had reported and testified against her husband, or just passed the information to the government or other helpline centres, then it is very possible that her life could have been saved. Government helpline centres need to work to win the trust of the people as distrust of government institutions and the justice system is a major contributor to this social problem. Reconciliatory gestures from the police, rapid justice delivery, transparent judiciary procedures, procedural ease for complaint registration have been considered effective for rebuilding the trust between police and community [16]. Nepal police in 2014 and 2016 have started reconciliatory gestures as “service with smile” and “police, my friend”. Spreading awareness of cases in remote areas and instilling a sense of responsibility in the locals should be initiated by the government with the help of domestic violence activists to prevent unreported cases.

## Figures and Tables

**Table 1:** Helpline services for DV in Nepal, governmental and non-governmental organizations [15]

Name	Hotline number
Government hotline	1234
Armed police force	1114
Nepal police rescue number	1113
Ministry of Women, Children, and Social Welfare (MOWCSC)	1618014200082

National Women Commission (NWC)	1145
Transcultural Psychosocial Organization (TPO)	16600102005
Women’s Rehabilitation Center (WOREC)	16600178910
Shakti Samuha	16600111117
